# Pathological Traits and Interpersonal Difficulties in Depressed Older Adults: Clinical versus Community Sampling

**DOI:** 10.1093/geroni/igab046.3239

**Published:** 2021-12-17

**Authors:** George Lederer, David Freedman, Lauren Atlas, Shira Kafker, Ira Yenko, Angel Mak, Dimitry Francois, Richard Zweig

**Affiliations:** 1 Jacobi Medical Center, Scarsdale, New York, United States; 2 Lenox Hill Hospital, Brooklyn, New York, United States; 3 New York-Presbyterian/Columbia University Irving Medical Center, NEW YORK, New York, United States; 4 Yeshiva University, Bronx, New York, United States; 5 NY, White Plains, New York, United States

## Abstract

Personality pathology, represented by high neuroticism and low agreeableness in the Five Factor Model of Personality, has been identified as a predictor of depression in mixed-age samples and preliminary studies of older adults. Research on older people, however, has not examined the differential impact of pathological personality traits and processes on depression or examined them across treatment settings. This secondary analysis examined personality traits and processes as predictors of depression, evaluated the moderating effect of interpersonal problems, and assessed stratification of these personality variables across community and clinical settings. Older adults (N=395) ranging in age from 55 to 99 (M = 72.06; SD = 10.10) from inpatient psychiatric, outpatient medical, and community settings completed self-report measures of personality traits (NEO-FFI Agreeableness and Neuroticism), processes (Inventory of Interpersonal Problems), and depression (GDS-30). Higher neuroticism predicted worsened depressive symptoms (β = .765, p < .001), as did lower agreeableness (β = -.163, p = .002) and more interpersonal problems (β = .459, p < .001). Findings partially supported the stratification of personality traits and processes by setting. Interpersonal problems moderated neither the neuroticism-depression or agreeableness-depression relationships. Personality traits and processes predict depression in older adults across care settings but do not significantly interact. Levels of pathological traits and processes vary across community and clinical settings.

